# The Type I interferon antiviral gene program is impaired by lockdown and preserved by caregiving

**DOI:** 10.1073/pnas.2105803118

**Published:** 2021-07-16

**Authors:** Steven W. Cole, John T. Cacioppo, Stephanie Cacioppo, Kyle Bone, Laura A. Del Rosso, Abigail Spinner, Jesusa M. G. Arevalo, Thomas P. Dizon, John P. Capitanio

**Affiliations:** ^a^Department of Psychiatry and Biobehavioral Sciences, David Geffen School of Medicine, University of California, Los Angeles, CA 90095;; ^b^Division of Hematology-Oncology, Department of Medicine, David Geffen School of Medicine, University of California, Los Angeles, CA 90095;; ^c^Norman Cousins Center, David Geffen School of Medicine, University of California, Los Angeles, CA 90095;; ^d^Jonsson Comprehensive Cancer Center, David Geffen School of Medicine, University of California, Los Angeles, CA 90095;; ^e^Center for Cognitive and Social Neuroscience, University of Chicago, Chicago, IL 60637;; ^f^Department of Psychiatry and Behavioral Neuroscience, University of Chicago, Chicago, IL 60637;; ^g^California National Primate Research Center, University of California, Davis, CA 95616;; ^h^Department of Psychology, University of California, Davis, CA 95616

**Keywords:** social genomics, infectious disease, public health, social behavior, social epidemiology

## Abstract

Previous research has linked perceived social isolation (loneliness) to reduced antiviral immunity, but the immunologic effects of the objective social isolation imposed by pandemic “shelter in place” (SIP) policies is unknown. We assessed the immunologic impact of SIP by relocating 21 adult male rhesus macaques from 2,000-m^2^ field cage communities of 70 to 132 other macaques to 2 wk of individual housing in indoor shelters. SIP was associated with 30% to 50% reductions in all circulating immune cell populations (lymphocytes, monocytes, and granulocytes), down-regulation of Type I interferon (IFN) antiviral gene expression, and a relative up-regulation of CD16^−^ classical monocytes. These effects emerged within the first 48 h of SIP, persisted for at least 2 wk, and abated within 4 wk of return to social housing. A subsequent round of SIP in the presence of a novel juvenile macaque showed comparable reductions in circulating immune cell populations but reversal of Type I IFN reductions and classical monocyte increases observed during individual SIP. Analyses of lymph node tissues showed parallel up-regulation of Type I IFN genes and enhanced control of viral gene expression during juvenile-partnered SIP compared to isolated SIP. These results identify a significant adverse effect of SIP social isolation on antiviral immune regulation in both circulating immune cells and lymphoid tissues, and they suggest a potential behavioral strategy for ameliorating gene regulatory impacts (but not immune cell declines) by promoting prosocial engagement during SIP.

## Significance

“Shelter in place” (SIP) orders have been deployed to slow the spread of SARS-CoV-2, but they induce social isolation that may paradoxically weaken antiviral immunity. We examined the impact of 2-wk SIP on immune cell population dynamics and gene regulation in 21 adult rhesus macaques, finding 30 to 50% declines in circulating immune cells, decreases in antiviral gene expression, and increased inflammatory cells in blood and inflammatory gene expression in lymph nodes. Declines in antiviral gene expression (but not circulating immune cells) were blocked by the presence of a novel juvenile partner during SIP, suggesting a potential strategy for maintaining antiviral immunity during SIP by enhancing prosocial engagement.

The COVID-19 pandemic has highlighted the complex role of sociality in human health, as social contact constitutes an essential resource for human well-being (1, 2), optimal physiological function (3, 4), and longevity (5, 6) but also a medium for the spread of infectious disease (7, 8). Efforts to reduce the spread of SARS-CoV-2 by social distancing are associated with reduced COVID-19 disease rates per unit time (9–14) but also incur substantial psychological, social, cultural, medical, and economic costs (14–18). Most cost–benefit analyses of social distancing policies overlook the fact that social contact is also an essential resource for optimal immune function (19–21). As such, extreme social distancing measures such as extended “stay at home” or “shelter in place” (SIP) orders may paradoxically increase vulnerability to viral infection (conditional on viral exposure) even as they reduce the risk of viral exposure. Social influences on host resistance to viral infections have been documented in experimental viral challenge studies in humans (19, 22, 23) and nonhuman primates (24, 25). However, the relevance of those studies to current (and historically novel) (26) extended SIP policies is complicated by the fact that earlier research assessed host resistance effects of perceived social isolation (“loneliness”) and low social network density (“social ties”) rather than objective social isolation (i.e., absence of others). Previous analyses also failed to capture the effects of some incidental nonsocial SIP effects such as transition from free mobility to involuntary confinement and reduced exposure to outdoor and natural environments. Research has begun to map the neural and immunoregulatory pathways through which loneliness and low social ties can influence immune function (24, 27–30), including a sympathetic nervous system (SNS)-mediated Conserved Transcriptional Response to Adversity (CTRA) involving up-regulated transcription of proinflammatory genes and down-regulated transcription of innate antiviral genes (e.g., Type I interferons; IFNs), resulting in part from increased output of classical CD16^−^ monocytes from bone marrow and splenic hematopoiesis (31–35). By contrast, little is known about the neural or immunoregulatory impact of the involuntary social isolation and confinement characteristic of extended SIP.

Given the documented adverse effects of extended SIP policies during the COVID-19 pandemic (14–18), there is a great need to identify strategies for mitigating their unintended harms while maintaining their intended epidemiologic benefits (9, 10). Previous experimental studies of CTRA gene regulation have shown that prosocial engagement (36), caregiving (37), and generativity (38) can reduce expression of proinflammatory genes and increase expression of Type I IFN genes (i.e., reduce the CTRA profile). These prosocial modes of behavior are hypothesized to activate central nervous system (CNS) reward circuits that subsequently inhibit the CNS threat response systems that control peripheral SNS activity and CTRA gene expression (39–42). This hypothesis raises the possibility that adverse immunological effects of extended SIP might potentially be reduced by promoting prosocial engagement during SIP.

## Results

To assess the immunologic impact of SIP, we relocated 21 adult male rhesus macaques from 2,000-m^2^ (half-acre) field cages containing 70 to 132 other macaques to 2 wk of individual housing in 2.0 × 0.8 × 0.7 m indoor shelters and examined changes in circulating immune cell (white blood cell; WBC) subpopulations, Type I IFN innate antiviral gene regulation, and viral gene transcription. Individual shelters met all Institutional Animal Use and Care Committee, US Department of Agriculture, and US NIH guidelines for humane macaque husbandry, including the presence of enrichment objects, daily foraging enrichment, and auditory and olfactory access to conspecifics in the same room.

**Fig. 1.**
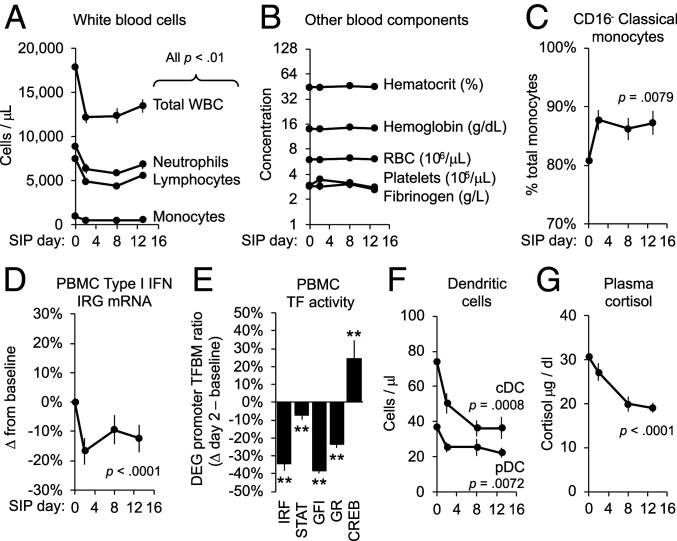
Effect of SIP on immune parameters. *n* = 21 community-housed adult macaques were relocated to individual indoor shelters for 14 d and assessed for (*A*) major leukocyte subsets, (*B*) red blood cells and other hematologic parameters, (*C*) relative prevalence of classical versus nonclassical monocytes, (*D*) per-cell expression of Type I IFN response genes in peripheral blood mononuclear cells (PBMC), (*F*) classical (cDC) and plasmacytoid (pDC) dendritic cells, and (*G*) plasma cortisol. (*E*) Change from baseline to SIP day 2 in bioinformatically inferred activity of IFN-related (IRF, STAT, GFI) and neuroendocrine-related (GR, CREB) transcription factors (TFs). Values: mean ± SE; *P* values: mixed effect linear model SIP day effect. **P < 0.01 difference from baseline.

As shown in Fig. 1, total immune cell (WBC) counts declined by an average 32% within the first 48 h of SIP (Fig. 1*A*; *F*(3, 20) = 37.13, *P* < 0.0001), whereas red blood cell counts, hematocrit, hemoglobin, platelet counts, and fibrinogen concentration remained stable (Fig. 1*B*). All major WBC subpopulations declined by 35 to 49% (Fig. 1*A*), including neutrophils (*F*(3, 20) = 6.34, *P* = 0.0034), lymphocytes (*F*(3, 20) = 14.29, *P* < 0.0001), and monocytes (*F*(3, 20) = 5.88, *P* = 0.0048). WBC reductions (leukopenia) persisted throughout the 2-wk SIP period with minimal abatement. Within the declining monocyte subpopulation, however, we observed multiple CTRA-characteristic immunoregulatory alterations including relative up-regulation of CD16^−^ classical monocytes (Fig. 1*C*; *F*(3, 20) = 5.24, *P* = 0.0079) and up-regulation of the CTRA gene expression profile (per-cell ratio of inflammatory versus Type I IFN response gene messenger RNA (mRNA): *F*(3, 3,546) = 3.01, *P* = 0.0291), the latter of which stemmed primarily from down-regulated expression of Type I IFN response genes (Fig. 1*D*; *F*(3, 1,969) = 6.92, *P* < 0.0001). Consistent with these alterations, promoter sequence-based bioinformatics analysis of the 1,804 gene transcripts that showed consistent change in average expression within the first 48 h of SIP (genes listed in Dataset S1) indicated reduced activity of transcription control pathways mediating expression of Type I IFNs (Fig. 1*E*; Interferon Response Factor/IRF: mean log_2_ ratio of transcription factor–binding motifs (TFBMs) in promoters of up- versus down-regulated genes: −0.607 ± 0.094, *z* = −6.45, *P* < 0.0001), IFN receptor signaling (STAT: −0.110 ± 0.033, *z* = −3.34, *P* = 0.0010), and development of the plasmacytoid dendritic cells (pDC) that constitute the primary source of Type I IFNs in circulating blood (43) (GFI: −0.700 ± 0.029, *z* = −23.98, *P* < 0.0001). Flow cytometry confirmed an average 19% reduction in circulating pDCs (Fig. 1*F*; *F*(3, 20) = 5.35, *P* = 0.0072) and 35% reduction in classical dendritic cells (cDCs; *F*(3, 20) = 8.43, *P* = 0.0008).

Stress-induced glucocorticoid release can reduce circulating WBC counts by shunting leukocytes out of blood and into other tissue compartments (44–47). Such effects do not appear to mediate the effects of SIP, however, because plasma cortisol concentrations did not increase during SIP but rather decreased progressively over 2 wk (Fig. 1*G*; *F*(3, 20) = 40.55, *P* < 0.0001). Stress can also alter circulating WBC numbers and CTRA gene expression via SNS catecholamine activation of β-adrenergic receptors that alter cell trafficking (48–50) and transcriptional regulation (31–35). Consistent with SNS activation, promoter-based bioinformatics analyses indicated increased activity of the CREB family of transcription factors that mediate β-adrenergic signaling (Fig. 1*F*; CREB: 0.316 ± 0.113, *z* = 2.80, *P* = 0.0056). Consistent with reduced circulating cortisol levels, parallel analyses indicated a significant decline in glucocorticoid receptor activity (Fig. 1*F*; GR: −0.391 ± 0.035, *z* = −11.20, *P* < 0.0001).

At the end of the 2-wk shelter period, animals were returned to their home outdoor field cages for 4 wk, during which all SIP-impacted immune parameters returned to baseline values (*SI Appendix*, Table S1).

### Social Buffering.

To determine whether prosocial engagement might buffer the immunoregulatory impact of SIP isolation, the same adult male macaques were subject to a second 2-wk SIP accompanied by a novel (unrelated) juvenile companion macaque (following an established conspecific caregiving protocol for abating effects of prolonged social isolation) (51). The 0.5- to 1.0-y-old male macaques were transferred to an individual shelter adjacent to each adult male, and a divider between the two shelters was subsequently removed to allow continuous interaction throughout the 2-wk SIP period. In all other respects the sheltering protocol was identical to the previous 2-wk isolated SIP. During juvenile-partnered SIP, adult macaques spent 23% of their time directly interacting with juveniles (e.g., grooming, contact, play), 51% of their time in the same cage, and 26% apart from the juvenile. The adults also showed a 54% reduction in abnormal behavior relative to isolated SIP (e.g., huddling, lying on floor, hanging on shelter walls; mean 186 ± 45 s per 1,200-s observation period versus 403 ± 68 during isolated SIP; *F*(1,20) = 27.05, *P* < 0.0001) and complementary increases in species-typical patterns of physical locomotion (+37%; 89 ± 22 versus 65 ± 22; *F*(1,20) = 21.76, *P* < 0.0001), sitting at rest (+29%; 879 ± 44 versus 680 ± 57; *F*(1,20) = 10.57, *P* = 0.0040), and ongoing exploratory behavior despite the arrival of a novel human intruder (a commonly employed measure of threat sensitivity; +267%; 2.64 ± 1.08 exploration events per 60-s observation period versus 0.72 ± 0.32; *F*(1,20) = 6.78, *P* = 0.0170).

**Fig. 2.**
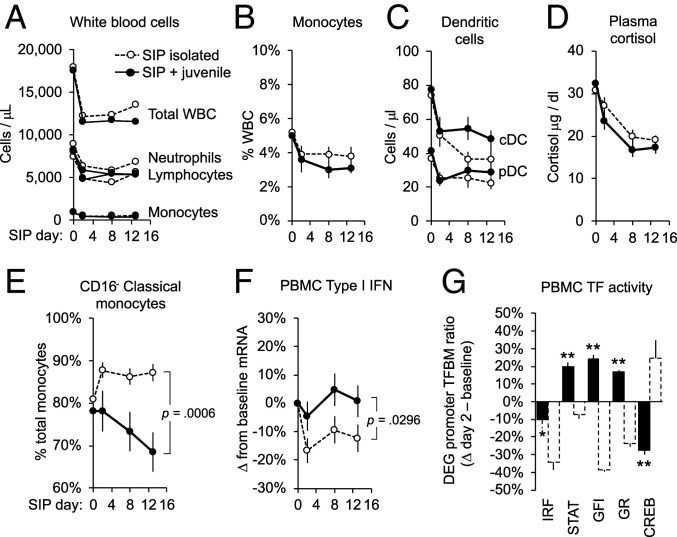
Social buffering of SIP effects. *n* = 20 adult macaques underwent a second 14-d SIP cycle paired with a juvenile macaque (solid lines/filled symbols). Effects were compared to isolated SIP trajectories (dashed lines/open symbols) for (*A*) major WBC subsets, (*B*) total monocytes, (*C*) dendritic cells, (*D*) plasma cortisol, (*E*) relative prevalence of classical versus nonclassical monocytes, (*F*) per-cell expression of Type I IFN response genes, and (*G*) RNA sequencing–based bioinformatic indications of IFN- related transcription factors (TFs: IRF, STAT, GFI) and neuroendocrine-related transcription factors (TFs) (GR, CREB). Values: mean ± SE; *P* values: SIP mode (isolated/paired) × SIP day interaction. *P < 0.05, **P < 0.01 difference from baseline.

As shown in Fig. 2, SIP with a juvenile conspecific (solid symbol/solid lines) did not significantly abate either WBC declines in general (Fig. 2*A*) or declines in monocytes and dendritic cells (Figs. 2 *B* and *C*). However, juvenile partnering did abate SIP effects on CTRA-characteristic immunoregulatory parameters including, 1) up-regulation of the classical monocyte subset (which now decreased from pre-SIP baseline, rather than increasing as observed in isolated SIP; Fig. 2*E*; SIP day × SIP mode interaction: *F*(3, 20) = 8.81, *P* = 0.0006); 2) down-regulation of Type I IFN gene expression (which now showed no significant decline from pre-SIP baseline; Fig. 2*F*; SIP day × SIP mode interaction contrast: *F*(3, 3,981) = 2.99, *P* = 0.0296); 3) down-regulation in bioinformatic indications of IFN-related transcription control pathways (which were all either quantitatively abated or fully reversed in analyses of 2,189 gene transcripts that showed consistent change in average expression from baseline to day 2 of juvenile-partnered SIP; Dataset S2; Fig. 2*G*; IRF: −0.156 ± 0.075, *z* = −2.09, *P* = 0.0382; STAT: 0.262 ± 0.029, *z* = 8.97, *P* < 0.0001; GFI: 0.312 ± 0.028, *z* = 11.35, *P* < 0.0001); and 4) bioinformatic indications of CREB activation (which now declined from pre-SIP baseline; Fig. 2*G*; −0.465 ± 0.064, *z* = −7.28, *P* < 0.0001). Parallel analyses indicated increased GR activity (0.225 ± 0.015, *z* = 15.65, *P* < 0.0001).

### Lymphoid Tissue Impact.

**Fig. 3.**
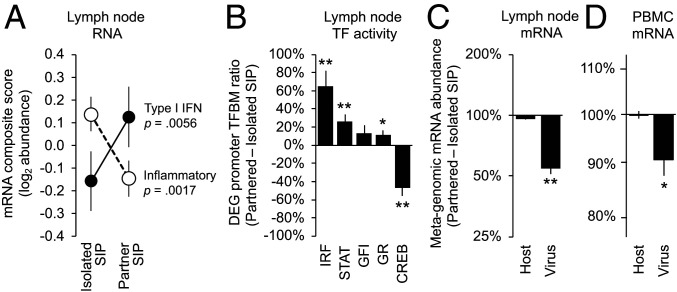
Effects on lymphoid tissue and viral activity. Lymph nodes were biopsied from *n* = 19 macaques at day 14 of isolated and juvenile-partnered SIP and assessed for (*A*) expression of proinflammatory and Type I IFN response genes, (*B*) RNA sequencing–based bioinformatic indications of change IFN-related (IRF, STAT, GFI) and neuroendocrine-related (GR, CREB) transcription factors (TFs), and (*C*) metagenomic sequence analysis of viral and metazoan (host/macaque) transcript abundance. (*D*) Parallel metagenomic sequence analysis of viral and metazoan (host/macaque) transcript abundance in PBMC. Values: mean ± SE; *P* values: mixed effect linear model SIP mode effect; *P < 0.05, **P < 0.01.

To assess the relevance of SIP-related changes in circulating WBC gene regulation for the lymphoid tissue environments in which leukocytes initiate adaptive antiviral immune responses (52), we biopsied axillary lymph nodes from each macaque at the end of each 2-wk shelter period. (Lymph nodes were not collected at pre-SIP baselines to avoid any immunologic impact of surgery during the SIP period.) Compared to lymph nodes collected after isolated SIP, those collected after juvenile-partnered SIP showed an 18% reduction in inflammatory gene expression (Fig. 3*A*; *F*(1, 18) = 13.54, *P* = 0.0017) and a 22% increase in Type I IFN response gene expression (Fig. 3*A*; *F*(1, 18) = 9.87, *P* = 0.0056). Promoter-based bioinformatic analysis of 884 gene transcripts showing consistent difference in expression following juvenile-partnered versus isolated SIP (Dataset S3) indicated increased activity of IFN-related transcription factors (Fig. 3*B*; IRF: 0.724 ± 0.144, *z* = 5.04, *P* < 0.0001; STAT: 0.332 ± 0.092, *z* = 3.63, *P* = 0.0004; and nonsignificant increase in GFI: 0.724 ± 0.144, *z* = 1.64, *P* = 0.1025) accompanied by reduced activity of CREB (−0.929 ± 0.235, *z* = −3.95, *P* = 0.0001) and increased activity of the GR (0.149 ± 0.072, *z* = 2.08, *P* = 0.0388).

### Antiviral Impact.

To determine how the immunoregulatory alterations associated with SIP might impact host response to viral infection, we conducted metagenomic RNA sequencing of lymph node tissues to quantify the relative abundance of host-derived (metazoan) and viral-derived gene transcripts (53). Compared to lymph nodes collected after isolated SIP, those collected after juvenile-partnered SIP showed a 45% reduction in viral gene transcripts as a fraction of total lymph node RNA abundance (Fig. 3*C*; −0.264 ± 0.026 log_10_ viral RNA reads per million total RNA reads, *F*(1, 18) = 99.40, *P* < 0.0001). To determine whether similar effects occur specifically for lymphotropic viruses, we conducted parallel metagenomic analyses of WBC RNA sequences collected at pre-SIP baseline and SIP day 2. Viral transcript abundance was 29-fold lower in circulating WBCs relative to lymph nodes, but results continued to show a 9.8% reduction in virus-derived gene transcripts during juvenile-partnered SIP (Fig. 3*D*; −0.046 ± 0.018, *F*(1, 20) = 6.45, *P* = 0.0196) whereas no significant reduction occurred during isolated SIP (−0.022 ± 0.020, *F*(1, 20) = 1.23, *P* = 0.2801).

## Discussion

Pandemic-style SIP induced rapid and persistent immunoregulatory alterations in rhesus macaques, including 30% to 50% reductions in circulating immune cell populations and CTRA-characteristic down-regulation of innate antiviral activity (Type I IFN response genes) and relative up-regulation of classical monocytes. These effects emerged within 48 h of “lockdown,” persisted for at least 2 wk, and abated within 4 wk of return to baseline social conditions. Provision of a novel juvenile partner during a subsequent round of SIP blunted CTRA-characteristic immunoregulatory dynamics (but not leukopenia), resulting in down-regulation of classical monocytes, increased Type I IFN gene expression, and preservation of antiviral gene regulation (IRF, STAT, GFI). Analyses of lymph nodes collected at the end of each SIP period showed parallel up-regulation of Type I IFN response genes and transcription control pathways following “lockdown with caregiving” compared to “lockdown alone.” Metagenomic sequencing confirmed the functional significance of changes in host antiviral gene transcription, documenting reduced viral gene expression during juvenile-partnered SIP relative to isolated SIP in both lymph nodes and circulating immune cells. These results identify significant reductions in host antiviral activity in both the circulating leukocyte pool available for recruitment into infected tissues and the lymphoid tissue leukocyte pool available to initiate adaptive immune responses (e.g., antibody and cytotoxic T cell production) during 2 wk of SIP. These results also suggest a potential strategy for ameliorating the CTRA-characteristic gene regulatory impacts (but not circulating leukopenia) by promoting prosocial engagement during SIP. The implications of these immunoregulatory dynamics for symptomatic viral disease remain to be quantified in future research, but the substantial immunobiological and virological impacts observed here suggest a need for such studies.

The distinct effects of juvenile partnering on SIP-induced leukopenia (immune cell declines) and CTRA gene regulation (reduced antiviral activity and increased classical monocytes) imply the existence of at least two distinct biological mechanisms for the observed array of effects. Consistent leukopenia indicates that SIP is physiologically stressful regardless of partner status. The magnitude and consistency of leukopenia across all major leukocyte subsets (including dendritic cells) might be hypothesized to stem from the leukopenic effects of stress-induced glucocorticoid release from the hypothalamus–pituitary–adrenal axis (44–46). However, this mechanism is unlikely to mediate the observed effects because, 1) circulating glucocorticoid levels declined substantially over the 2-wk SIP periods [as previously observed (54, 55) and likely due to reduced exposure of adult males to dominance-related agonistic interactions in group housing (56, 57)] rather than increasing as would account for leukopenia (47); and 2) glucocorticoids selectively increase circulating neutrophil numbers (47), whereas neutrophil numbers declined in parallel with lymphocyte and monocyte counts during both SIP cycles. Global leukopenia is also inconsistent with the previously observed effects of acute SNS activation in up-regulating circulating neutrophil, monocyte, and lymphocyte numbers (48–50) rather than downregulating them as observed here. As such, the physiological mechanisms underlying SIP-associated leukopenia remain to be defined, with changes in leukocyte development, cell death, and trafficking patterns representing key targets for future research.

By contrast, SIP-induced changes in SNS activity do provide a parsimonious explanation for the isolation-specific reductions in Type I IFN gene regulation, increases in CD16^−^ classical monocyte prevalence (relative to CD16^+^ nonclassical monocytes), and up-regulated viral activity in lymphoid tissue and circulating immune cells. These changes are all consistent with the SNS-induced CTRA gene regulation program (31–35), which is known to be activated by adverse social conditions in macaques (24, 25, 58) and has previously been linked to impaired control of viral infections (24, 25). Consistent with this hypothesis, 1) promoter-based bioinformatics analyses indicated up-regulation of the CREB transcription control pathway that mediates β-adrenergic signaling from the SNS (59), and 2) CREB activity was reduced by the presence of a juvenile partner during SIP, in parallel with other CTRA-characteristic regulatory dynamics. Reductions in lymph node CREB activity during juvenile-partnered SIP may also stem from reductions in stress-mediated neural activity and structural arborization of sympathetic nerve fibers within lymphoid tissues (25, 58). Parallel increases in GR activity during juvenile-partnered SIP may stem from reduction in SNS-mediated GR desensitization associated with stress myelopoiesis (33, 34, 60). However, definitive support for an SNS mechanism will require experimental inhibition of sympathetic nerve activity or β-adrenergic signaling to abrogate SIP effects on CTRA gene regulation.

The CTRA-inhibitory effects of juvenile-partnered SIP are consistent with previous research showing that caregiving, generativity, and other modes of prosocial engagement can down-regulate CTRA gene expression in humans (36–38). These effects are hypothesized to be mediated by activation of CNS reward circuits that laterally inhibit activity of CNS threat response systems (41, 42, 61) and prosocial engagement of the parasympathetic nervous system (62), both of which act to reduce basal sympathetic tone and the β-adrenergic signaling pathways that mediate CTRA gene expression (31, 32). Consistent with reduced CNS threat processes, adult macaques displayed lower rates of distress-related behavior (e.g., huddling, hanging, lying) and higher rates of species-typical behavior (e.g., seated rest, normal mobility, cage exploration) during juvenile-partnered SIP compared to isolated SIP. However, it is possible that partnered SIP also acts in ways that do not involve prosocial motivation per se, such as effects of physical contact, motor activity, or microbial exposures. Given the marked impacts documented here, the isolated versus partnered-SIP paradigm may be a useful experimental system for broader analyses of the pathways through which social exposures buffer biological responses to adversity. Social exposures can also generate physiological costs resulting from social burdens (63) as previously observed when older adult macaques were paired with multiple juvenile partners (64). Adverse effects may be amplified by competing demands (e.g., from work, education, additional social partners, etc.) or more extended SIP durations than studied here and could be clarified by parametric variation of the present paradigm (e.g., > 2-wk duration; multiple partners; novel versus familiar juvenile partners, adult peers, biological relatives; competing engagements; etc.).

The present results suggest a psychobiological mechanism for the observation that parents and others who cohabitate with children show reduced vulnerability to some viral diseases (65–68). Studies have documented reduced risk of COVID-19 (but not SARS-CoV-2 exposure/infection) among adults living in households with children (65–67). These effects have been interpreted as stemming from immune shielding (i.e., children’s robust antiviral responses reduce the intensity of viral transmission to household adults and thereby reduce the incidence of symptomatic disease). The present analyses suggest an alternative mechanism whereby caregiving adults may show physiologically mediated alterations in immune cell gene regulation that promote host resistance to viral disease (e.g., elevated Type I IFN activity). Such effects would be consistent with previous viral challenge studies that document reduced risk of respiratory virus infections and symptomatic disease among parents (including those whose children have already left home) (68).

This research is subject to several limitations, including imperfect recapitulation of human “stay at home” policies (e.g., macaques were confined in novel indoor cages rather than a familiar “home”), a 2-wk duration (effects may differ for longer SIP durations characteristic of human public health mandates), assessment of adult males only (which might underestimate the protective effects of “caregiving” if such responses are more frequent or pronounced in females) (69), and a relatively simple partnering protocol (different effects may occur with different partner numbers or characteristics, or with greater competing demands). SIP also affects nonsocial processes (e.g., mobility, natural environment exposure), and the immunologic effects observed here cannot be attributed purely to social deprivation. Partnered SIP always followed isolated SIP in this study, which might confound partner status with habituation (although recurrent leukopenia shows any habituation to be partial at best). This study does not contain any direct measures of viral disease (tissue pathology, illness symptoms) or host resistance to de novo infection, and the health significance of the observed effects remains to be defined in future research.

SIP impaired host control of viral infections in this study, but these results do not imply that the costs of SIP outweigh its benefits. Policy analyses of disease prevalence capture the net effect of host resistance costs and viral exposure benefits, and substantial observational data have linked social distancing policies in general to reduced viral disease rates per unit time (9, 10). However, among all distancing policies examined, SIP and extended “stay at home” orders appear to have the weakest net benefit (i.e., above and beyond more targeted business closures, school closures, and restrictions on large gatherings) (9, 11–14). The present results suggest that the relatively modest epidemiologic benefits of SIP policies may stem in part from their unrecognized costs in undermining host resistance to viral infection even as they reduce the probability of viral exposure. To the extent that SIP is retained as a policy response (e.g., due to political demand), it may be possible to enhance SIP’s epidemiologic benefit by altering the mode and conditions of sheltering to maximize caregiving opportunities and other prosocial engagements. Mapping the psychological and biological mechanisms involved may also suggest new policy, behavioral, or pharmacologic strategies for controlling the immunological impacts of protracted social isolation and thus help evolve more sustainable and effective disease mitigation strategies for social control of pandemic infectious diseases.

## Methods

A total of 21 adult male rhesus macaques were relocated from their home 2,000-m^2^ field cages containing 70 to 132 other macaques to 2 wk of individual housing in 2.0 × 0.8 × 0.7 m indoor quarters for adult male rhesus macaques at the California National Primate Research Center. Individual quarters comprised two standard individual housing cages (1.0 m W × 0.8 H × 0.7 m D) connected by an opened door and met all Institutional Animal Care and Use Committee, US Department of Agriculture, and US NIH guidelines for humane macaque husbandry, including the presence of enrichment objects, daily foraging enrichment, and auditory and olfactory access to conspecifics in the same room. Relocation to individual quarters occurred between 8:00 and 8:45 AM. A 7.5 mL venipuncture blood sample was obtained at 3:00 PM 1 wk prior to and 2, 8, and 13 d after relocation. Distress-related and species-typical (nondistressed) behaviors were quantified by ethogram scoring of 5-min videotaped behavioral samples collected four times per day between 9:00 and 11:00 AM from each animal at day 1, 2, 7, 8, 12, and 13 and separate observations during a “human intruder” behavioral challenge at 3:00 PM on day 9 (see details in *SI Appendix*). On day 14, each animal underwent an axillary lymph node biopsy and subsequently recovered in the hospital for ≥5 d before return to their home field cage.

Approximately 1 mo after return to their home field cage, each macaque was again relocated to the same individual shelter, which now contained a 0.5- to 1.0-y-old novel (unrelated) male macaque (following previous “therapy monkey” protocols for socially isolated macaques) (51). With the exception of juvenile partner pairing, all other aspects of the sheltering protocol were identical to those of the previous round of isolated sheltering.

All procedures were approved by the Institutional Animal Care and Use Committee of the University of California, Davis.

### Hematology, Immune Cell, and Hormone Analysis.

Detailed analytic methods are presented in *SI Appendix*, *Detailed Methods*. Briefly, each blood sample was assayed by automated complete blood count with differential; flow cytometric enumeration of major leukocyte subsets, classical (CD16^−^), and nonclassical (CD16^+^) monocytes, and CD3^−^/CD20^−^/HLA-DR^+^/CD123^+^ pDC and CD3^−^/CD20^−^/HLA-DR^+^/CD1c^+^ cDC; chemiluminescent immunoassay of plasma cortisol; and mRNA sequencing of peripheral blood mononuclear cells (PBMCs) to generate genome-wide transcriptional profiles (70). Transcriptional profiles were analyzed to quantify expression of prespecified sets of genes involved in Type I IFN, proinflammatory, and CTRA gene regulation (71). Activity of Type I IFN- and pDC-related transcription control pathways (IRF, STAT1, GFI) and SNS- and GR-related transcription control pathways (CREB, GR) was also assessed using promoter-based bioinformatics analyses of all gene transcripts (genome-wide) found to show consistent up-regulation versus down-regulation from baseline to SIP day 2 (blood cells) or from isolated to juvenile-partnered SIP (lymph nodes). Viral and metazoan gene expression were quantified by metagenomic RNA profiling (53).

### Data Analysis.

Hematology, flow cytometry, cortisol, and leukocyte RNA data were analyzed by mixed effect linear models (SAS 9.4 PROC MIXED) specifying fixed effects of SIP day (baseline, day 2, 8, and 13), SIP mode (isolated versus juvenile-partnered), a SIP day × SIP mode interaction, and a random effect of subject (animal) with a fully saturated (unstructured) variance–covariance matrix to account for heteroscedasticity and correlation among residuals. For parameters assessed once per SIP cycle (behavior, lymph node RNA), parallel mixed effect linear models analyzed fixed effects of SIP mode (isolated versus juvenile partnered).

## Supplementary Material

Supplementary File

Supplementary File

Supplementary File

Supplementary File

## Data Availability

Anonymized RNA profiling data have been deposited in Gene Expression Omnibus (GSE174065) (70).
