# Supplementary material for: The Type I interferon antiviral gene program is impaired by lockdown and preserved by caregiving
Source: Proc Natl Acad Sci U S A. 2021 Jul 16;118(29):e2105803118. doi: 10.1073/pnas.2105803118 (PMC8307615; doi:10.1073/pnas.2105803118)
Supplement: Supplementary File [file pnas.2105803118.sapp.pdf]

PNAS
www.pnas.org

Supplementary Information for

The Type I interferon antiviral gene program is impaired by lockdown and preserved by caregiving

Steven W. Cole, John T. Cacioppo, Stephanie Cacioppo, Kyle Bone, Laura A. Del Rosso, Abigail Spinner, Jesusa M. G. Arevalo, Thomas P. Dizon, and John P. Capitanio

Corresponding author: Steven W. Cole

Email: steve.cole@ucla.edu

This PDF file includes:

Supplementary text

SI Tables S1 and S2

SI Figure S1

SI References

Other supplementary materials for this manuscript include the following:

Datasets S1 to S3

Supplementary Text

Detailed Methods

Sample and procedures. 21 adult male rhesus macaques were relocated from their home half-acre field cages containing 70-132 other macaques to 2 wks of individual housing in 2.0 x 0.8 x 0.7 m indoor quarters for rhesus macaque husbandry at the California National Primate Research Center. Individual quarters comprised two standard individual housing cages (1.0 m W x 0.8 H x 0.7 m D) connected by an opened door, and met all Institutional Animal Care and Use Committee, U.S. Department of Agriculture, and U.S. National Institute of Health guidelines for humane macaque husbandry, including the presence of enrichment objects, daily foraging enrichment, and auditory and olfactory access to conspecifics in the same room. Relocation to individual quarters occurred between 8:00 and 8:45 AM. Two animals were relocated at a time, and were housed in adjacent cages that were out of visual contact with each other. 7.5 ml blood samples were obtained at 3:00 PM 1 wk prior to and 2, 8, and 13 d after relocation. Samples were collected via femoral venipuncture after manual restraint using the intra-cage squeeze mechanism. On d 14, each animal underwent an axillary lymph node biopsy and subsequently recovered in the hospital for ≥ 5 d before return to their home field cage.

Approximately 1 mo after return to their home field cage, each macaque was again relocated to the same individual shelter, which now contained a novel (unrelated) 0.5-1.0 yr-old juvenile macaque in 1 of the 2 linked cages. This procedure follows “therapy monkey” protocols previously used with socially isolated rhesus macaques (1). Juveniles were relocated by 8:00 AM, providing 15-30 min familiarization before arrival of the adult male study animal. Paired animals were monitored in person and by camera to ensure safety. With the exception of juvenile partner pairing, all other aspects of the sheltering protocol were identical to those of the previous round of isolated sheltering.

All procedures were approved by the Institutional Animal Care and Use Committee of the University of California, Davis.

Hematology, immune cell, and hormone analysis. Blood cell counts were determined by automated CBC with differential (Pentra 60C+). $CD14^{++}/CD16^{-}$ and $CD14^{+}/CD16^{++}$ monocyte percentages, $CD3^{-}/CD20^{-}/HLA\text{-}DR^{+}/CD123^{+}$ pDC and $CD3^{-}/CD20^{-}/HLA\text{-}DR^{+}/CD1c^{+}$ cDC percentages, and percentages of $CD3^{+}/CD4^{+}$ T cells, $CD3^{+}/CD8^{+}$ T cells, $CD20^{+}$ B cells, and $CD3^{-}/CD16^{+}CD56^{+}$ NK cells were determined by flow cyotmetry on a BD LSR Fortessa instrument (BD Biosciences). Assay antibodies were: CD3 PacBlue (BD-Biosciences 558124), CD4 BV605 (BD-Biosciences 562843), CD8 BV510 (BD-Biosciences 563919), CD20 Alexa700 (BD-Biosciences 560631), CD123 BV786 (BD-Biosciences 564196), CD14 FITC (BD-Biosciences 557153), CD16 PE-Cy7 (BD-Biosciences 557744), HLA-DR PerCP-Cy5.5 (BD-Biosciences 552764), and CD11c APC (BD-Biosciences 340714). Plasma cortisol was assayed by chemiluminescent immunoassay as previously described (2). Hematology, flow cytometry, and cortisol data were analyzed by mixed effect linear models (SAS 9.4 PROC MIXED) specifying a fixed effect of SIP Day (baseline, d 2, d 8, d 13; repeated measure), a random effect of subject (animal), and a fully saturated (unstructured) variance-covariance matrix to account for residual correlation and heteroscedasticity:

```
proc mixed method=ml;
     class AnimalID SIPDay;
     model Outcome = SIPDay / solution;
     repeated / subject=AnimalID type=UN;
     lsmeans SIPDay;
```

where AnimalID represents the subject, SIPDay represents the time point, and Outcome represents the biological parameter analyzed (e.g., WBC counts). Analyses comparing isolated vs. juvenile-partnered SIP included additional fixed effects of SIP Mode (isolated vs. juvenile-partnered; repeated measure) and a SIP Day x SIP Mode interaction:

```
proc mixed method=ml;
```

```
     class AnimalID SIPMode SIPDay;
     model Outcome = SIPDay|SIPMode / solution;
     repeated / subject=AnimalID type=UN;
     lsmeans SIPMode*SIPDay;
```

Similar analyses tested for recovery to baseline hematological values following isolated SIP by comparing pre-SIP baseline measures to measures collected at 4 wks post-SIP (which also served as the pre-SIP baseline for the subsequent juvenile-partnered SIP cycle):

```
proc mixed method=ml;
     class AnimalID SIPMode;
     model Outcome = SIPMode / solution;
     repeated / subject=AnimalID type=UN;
     lsmeans SIPMode;
```

Behavior. Distress-related and species-typical (non-distressed) behaviors were quantified by ethogram scoring of 5-min behavioral samples collected from each animal 4 times per d between 9:00 and 11:00 AM at d 1, 2, 7, 8, 12, and 13, resulting in a total of 20 min of data per observation day (120 min total) for each animal in each SIP cycle. Behavioral samples were videotaped (Sony HDR-CX305 Handycam) for later coding by a trained observer using the Observer software (3). The scorer used a standard ethogram for animals of this age and species (Table S2 below) and had demonstrated 91.5% agreement in tests of inter-observer reliability conducted prior to scoring. Behavioral categories scored included high-prevalence species-typical behaviors such as sitting at rest and normal physical locomotion (e.g., walking) as well as abnormal behaviors characteristic of threat response or distress (e.g., huddle, huddle-like, lie, sleep, hang, crouch, and motor stereotypy). Data were coded as seconds of duration for each scored category, and total durations of each category were summed for each animal on each day and averaged over the 6 observed days on each SIP cycle (after finding no significant SIP Day x SIP Mode interactions). Behavioral data on social threat sensitivity were also obtained in a Human Intruder challenge that has been described previously (4) and is commonly used in non-human primate studies (4-7) to assess behavioral inhibition, or the disruption of species-typical ongoing environmental exploratory behavior in response to an

ambiguous social stimulus. Human Intruder tests were conducted on d 9 of each SIP cycle, with the juvenile temporarily confined away from the adult male macaque in one of the two housing cages. Briefly, a human staff member stood in front of each cage for four 1-min trials, first presenting a face in profile from a distance far (~1 m) from the adult male macaque and then a near (~0.3 m), followed by staring directly at the adult male macaque from the two locations. Responses were recorded on video and coded using the Observer software and an ethogram similar to Table S2 and detailed in (4). Measures of environmental-exploratory behavior were summarized across the four trials after preliminary analyses found no SIP Mode x Trial interactions. All behavioral data were log-transformed to address skew and heteroscedasticity and mixed effect linear models analyzed fixed effects of SIP Mode (isolated vs. juvenile-partnered; repeated measure) and random effects of subject with an unstructured variance-covariance matrix specified to account for residual heteroscedasticity and correlation across repeated measurements (SAS 9.4 PROC MIXED):

```
proc mixed method=ml;
     class AnimalID SIPMode;
     model Outcome = SIPMode / solution;
     repeated / subject=AnimalID type=UN;
     lsmeans SIPMode;
```

Blood transcriptome profiling. Peripheral blood mononuclear cells (PBMC) were isolated from venipuncture blood samples by standard Ficoll density gradient centrifugation, stored at -70C, and subsequently assayed by mRNA sequencing in a single batch. Total RNA was isolated (Qiagen RNeasy), tested for suitable mass (> 50 ng by Pico Green RNA) and integrity (Agilent TapeStation RNA Integrity Number > 3), and 300 ng aliquots were assayed using a high-efficiency 3' mRNA counting assay (Lexogen QuantSeq 3' FWD) performed on an Illumina HiSeq 4000 instrument following the manufacturers' standard protocols. Assays targeted > 10 million 65-base single-strand reads per sample (achieved mean 13.1 million), each of which were mapped to the NCBI reference macaque genome (Mmul_10) using the STAR aligner (8)

(average mapping rate 95.6%). Transcript counts per million mapped reads (9) were floored at 1 TPM (to suppress spurious variability), $\log_2$-transformed, and analyzed by mixed effect linear models as described below to quantify change from baseline to SIP days 2, 8, and 13 and any difference in such effects as a function of SIP Mode. Ancillary analyses additionally included flow cytometric proportions of $CD4^+$ and $CD8^+$ T cells, B cells, NK cells, monocytes, pDCs, and cDCs to ensure results were independent of cell subset distributions (i.e., reflect per-cell differences in gene expression). Initial analyses examined pre-specified sets of 19 gene transcripts used previously (10) to quantify inflammatory gene regulation (*CXCL8*, *FOS*, *FOSB*, *FOSL1*, *FOSL2*, *IL1A*, *IL1B*, *IL6*, *JUN*, *JUNB*, *JUND*, *NFKB1*, *NFKB2*, *PTGS1*, *PTGS2*, *REL*, *RELA*, *RELB*, *TNF*), 32 gene transcripts used previously (10) to quantify Type I interferon response and pDC activation (*IFI16, IFI27, IFI27L2, IFI30, IFI35, IFI44, IFI44L, IFI6, IFIH1, IFIT1, IFIT2, IFIT3, IFIT5, IRF2, IRF7, IRF8, JCHAIN, MX1, MX2, OAS1, OAS2, OAS3, OASL*; and omitting the previously used *IFITM5* and *IFNB1* due to minimal expression levels in this data set at SD < .5 $\log_2$ abundance) and 51 gene transcripts used previously (10) to quantify the CTRA profile (the union of the 19 pro-inflammatory and 32 Type I IFN indicator gene listed above, with the later sign-inverted to reflect their inverse contribution to the CTRA profile). Transcript abundance values were standardized to mean 0 and SD 1 within gene to facilitate maximum likelihood estimation and analyzed by mixed effect linear models (SAS 9.4 PROC MIXED) specifying fixed effects of Gene (repeated measure) and SIP Day (baseline, d 2, d 8, d 13; repeated measure), and a random effect of subject (animal):

```
proc mixed method=ml;
     class Gene AnimalID SIPDay;
     model Expression = Gene SIPDay / solution;
     random intercept / subject=AnimalID;
     contrast 'SIPAvgVsBL' SIPDay -3 1 1 1;
     lsmeans SIPDay;
```

where Gene indexes specific indicator transcripts, AnimalID indexes subject animals, SIPDay indexes time point, and Expression represents standardized $\log_2$ transcript abundance values.

Analyses comparing isolated vs. juvenile-partnered SIP included additional fixed effects of SIP Mode (isolated vs. juvenile-partnered; repeated measure) and a SIP Day x SIP Mode interaction:

```
proc mixed method=ml;
     class Gene AnimalID SIPMode SIPDay;
     model Expression = Gene SIPMode|SIPDay / solution;
     random intercept / subject=AnimalID;
     lsmeans SIPMode*SIPDay;
```

Follow-up analyses quantified activity of transcription control pathways involved in Type I IFN response using TELiS promoter-based bioinformatics analyses (11) of all gene transcripts (genome-wide) that showed consistent change in expression from baseline to SIP d 2. TELiS infers transcription factor activation based on asymmetry in the prevalence of transcription factor-binding motifs (TFBMs) in the core promoter sequences of genes found to be up-regulated vs. down-regulated, testing the observed ratio of TFBMs in up- vs. down-regulated gene promoters for statistically significant departure from the null hypothesis ratio of 1. Genes were screened into TELiS analyses based on a measure of consistent change from baseline defined as the ratio of the average change in expression to the standard error of that change as estimated in a standard linear statistical model, with the threshold for consistency arbitrarily selected to match a nominally significant *t* ratio at p = .05). This consistency-based gene-screening criterion optimized the balance of sensitivity (maximizing the number of active promoter sequences available for analysis), specificity (maximizing signal-to-noise ratio in linear model estimates of differential expression), and symmetry (similar numbers of up- and down-regulated genes, to mitigate differential precision in numerator and denominator promoter prevalence estimation) for TPM-normalized data. In ancillary sensitivity analyses, we also examined the effect of an alternative input gene-screening criteria and found similar substantive results when TELiS was applied to all gene transcripts that showed > 2-fold change in average expression from baseline to SIP d 2 after TPM values were additionally normalized to

standardize expression of 11 established reference genes (12) and thereby reduce technical variability and enhance sensitivity, specificity, and symmetry while minimizing false-negative bias associated with *p*-value- or False Discovery Rate *q*-value-based screening (13-16). TELiS analysis of Type I IFN and neural/endocrine transcriptional control pathways used TRANSFAC position-specific weight matrices to assess interferon response factors (V$ISRE_01), STAT1 (V$STAT_01), and GFI1 (V$GFI1_01) as well as β-adrenergic signaling via CREB (V$CREB_01, V$TAXCREB_01) and hypothalamus-pituitary-adrenal axis signaling via the glucocorticoid receptor (V$GR_Q6). As in previous studies (10), analyses were conducted using 9 parametric combinations of core promoter sequence length (-300, -600, and -1000 to +200 nt relative to the RefSeq transcription start site) and TFBM detection stringency (TRANSFAC mat_sim information criteria of .80, .90, or .95), with the 9 resulting TFBM ratios $\log_2$ transformed and averaged for comparison with the null hypothesis value of 0 (i.e., $\log_2$-1-fold ratio, or no difference in TFBM prevalence in up- vs. down-regulated promoters). Statistical significance was assessed by z test, with the standard error of averaged $\log_2$ TFBM ratios estimated by bootstrap resampling of linear model residual vectors (17) to account for the effect of correlation among genes on stochastic variation in TFBM ratios. Beyond this pre-specified analysis of CTRA-related transcription factor activity, we did not attempt any other hypothesis-free exploratory/discovery analyses of genome-wide transcriptome differences because this study was not powered to generate reliable results from such analyses. Linear statistical model quantification of change in the expression of individual gene transcripts was applied only as a screening metric to exclude from TELiS analyses those genes that showed inconsistent response to SIP in TPM normalized data. (Statistical significance of TELiS TFBM ratios was tested using a separate bootstrap resampling analysis that did not involve gene-specific measures of statistical significance.) Screen-passing genes listed in Datasets S1-S3 provide qualitative characterization of transcriptome alteration and should not be interpreted as indicating quantitatively reliable change in expression of individual gene transcripts.

Impacts on viral gene regulation were assessed at baseline and SIP d 2 using the Kingdom Binner module of the Taxonomer meta-genomic mapping system (18) to quantify relative abundance of viral and metazoan RNA sequences, with mixed effect linear models analyzing $\log_{10}$ transcript abundance values as a function of Kingdom of origin (viral vs. metazoan; repeated measure), SIP Day (repeated measure), SIP Mode (repeated measure), and their interactions (repeated measures), as described above.

Lymph node transcriptome profiling. Biopsied axillary lymph nodes were flash frozen for storage at -70°C, thawed and dissociated to single cell suspensions as previously described (19), and subject to mRNA sequencing as described above for PBMC (with analyses omitting the SIP Day parameter, as lymph nodes were collected only at SIP d 14 in each round). Lymph node data were unavailable for 2 animals due to insufficient RNA recovery, yielding 19 animals for repeated measures analysis. Transcript counts per million mapped reads (9) were floored at 1 TPM (to suppress spurious variability), $\log_2$-transformed, and analyzed as described above using mixed effect linear models specifying fixed effects of SIP Mode (isolated vs. juvenile-partnered; repeated measure) and random effects of subject (animal) with an unstructured variance-covariance matrix to account for residual heteroscedasticity and correlation across repeated measurements (SAS 9.4 PROC MIXED). TELiS bioinformatics analyses were applied to genes showing >1.5-fold differential expression due to the lower number of transcripts showing >2-fold differential expression in lymph nodes compared to PBMC.

Supplemental Results

Figure S1: Effect of Isolated vs Juvenile-Partnered SIP on behavioral measures of threat sensitivity.

Table S1: Comparison of Pre-SIP 1 baseline with Post-SIP 1 follow-up (Pre-SIP 2 baseline)

Table S2: Ethogram for Adult Macaque Behavior

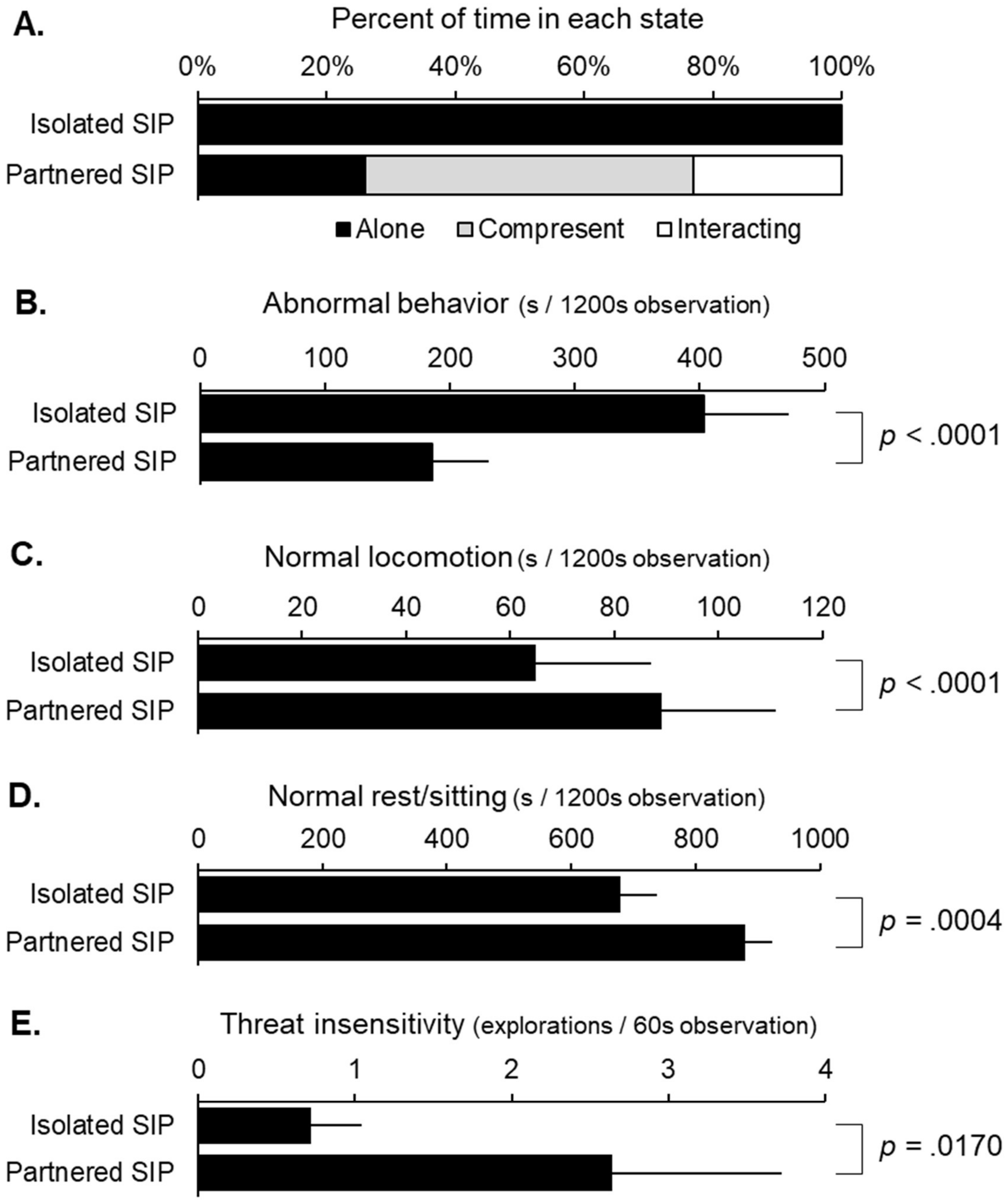

Figure S1. Behavior during isolated and juvenile-partnered SIP. Videotaped behavior samples were collected on N=20 adult macaque study subjects at d 1, 2, 7, 8, 12, and 13 of isolated and juvenile-partnered SIP (20 min/d; total 120 min/SIP cycle) and analyzed for (A.) time spent apart from, in the same cage as, and directly interacting with juvenile partner macaques, and relative prevalence of (B.) abnormal behaviors (e.g., huddling, lying on floor, hanging on shelter walls) and species-typical (C.) locomotion and (D.) seated rest. (E.) On d 9 of each SIP period adult study subject macaques were exposed to a novel human intruder who assumed 4 progressively more threatening postures and behavioral threat sensitivity was assessed by behavioral inhibition (i.e., reduction in normal environmental exploratory behavior observed during 4 60s observation periods). Values: mean ± SE. *p*-values: Mixed effect linear model SIP Mode effect.

Table S1: Comparison of Pre-SIP 1 baseline with Post-SIP 1 follow-up (Pre-SIP 2 baseline)

Biological parameter	SIP 1 baseline	SIP 1 4-wk follow-up / SIP 2 baseline	Difference *p*-value
WBC count (x1000 cells/uL)	17.9 (1.0)	17.6 (0.9)	0.7235
Neutrophil count (x1000 cells/uL)	8.9 (0.6)	8.0 (0.5)	0.2487
Lymphocyte count (x1000 cells/uL)	7.4 (0.6)	8.2 (0.5)	0.0952
Monocyte count (x1000 cells/uL)	0.9 (0.1)	0.8 (0.2)	0.4182
Hematocrit (%)	44.3 (0.6	44.3 (0.7)	0.9408
Hemoglobin (g/dL)	14.1 (0.2)	14.0 (0.3)	0.3815
RBC count (10^6/uL)	6.01 (0.08)	6.02 (0.09)	0.9119
Platelet count (10^5/uL)	2.98 (0.23)	3.21 (0.25)	0.4751
Fibrinogen (g/L)	290 (23)	262 (29)	0.4411
Classical monocytes (% total monocytes)	80.1 (2.1)	78.2 (2.3)	0.2521
Cortisol (ug/dL)	31.5 (2.0)	33.4 (1.9)	0.3602
CTRA gene expression (standardized log2 RNA)	-0.001 (0.041)	0.001 (0.041)	0.9096
Type I IFN gene expression (standardized log2 RNA)	-0.006 (0.066)	0.006 (0.066)	0.7156
Inflammatory gene expression (standardized log2 RNA)	0.003 (0.057)	-0.003 (0.057)	0.8501
Classical dendritic cells (cells/uL)	74.0 (8.7)	77.1 (10.2)	0.6615
Plasmacytoid dendritic cells (cells/uL)	36.7 (5.2)	41.5 (6.4)	0.3539

Values: mean (SE)

Table S2: Ethogram for Adult Macaque Behavior

ACTIVITY - Categories are mutually exclusive and exhaustive.

Active: Directed whole body movement.

Huddle: Sit with head at the same level of or lower than shoulders; arms and limbs huddle to the center of the body; no movement of the body or the four limbs for at least 2 sec; eyes open or if unable to determine whether the eyes are open. When huddling, the animal can yawn or scratch.

Huddle Like: Similar to Huddle state, except that here, arms and limbs positioned to the outside of the body. When in this state, the animal can yawn or scratch.

Lie: Relaxed posture with body resting on a horizontal surface for at least 2 seconds. Weight is not supported by limbs.

Sleep: In any position with eyes closed for at least 2 seconds. If eyes are obscured, then code default posture (i.e., if animal is sitting, code sit, etc.).

Sit: Hindquarters are on the perch or floor at least 2 seconds; includes shifting weight slightly one step.

Stand: Torso in a stationary position and weight is supported by one or more limbs on floor, or perch bar at least 2 seconds; can shift weight.

Hang: Holding onto ceiling or front mesh at least 2 seconds; all 4 limbs off of floor. If one limb on floor, or perch bar, score stand.

Crouch: Ventral surface close to floor at least 2 seconds; head at or below the level of the shoulders

Motor Stereotypy: Repetitive, patterned, or rhythmic movement at least 2 seconds (e.g., pacing, bouncing, head toss).

EVENTS

Approach: One animal moves into his pair-mate's cage.

Environmental Explore: Discrete manipulation by hand(s) or mouth with the physical environment or objects in the cage.

Self-Directed Stereotypy: Non-motor stereotypy such as salute/eye poke, eye covering, self-clasping, or self-directed pulling or pushing of any part of the body.

Present Groom: One animal intentionally presents his neck, belly, rump, back or side (flank) to another animal.

Present Sex: Rigid posture with rump and tail (if tail exists) elevated and oriented toward another individual, often preceded by a look and/or a vocalization.

SI References

1. Novak MA (1979) Social Recovery of Monkeys Isolated for the First Year of Life: II. Long-Term Assessment. *Developmental Psychology* 15(1):50-61.
2. Vandeleest J*, et al.* (2019) Social stability influences the association between adrenal responsiveness and hair cortisol concentrations in rhesus macaques. *Psychoneuroendocrinology,* 100:164–171.
3. Noldus L (1991) The Observer: a software system for collection and analysis of observational data. *Behavior Research Methods, Instruments, & Computers* 23:415-429.
4. Gottlieb DH & Capitanio JP (2013) Latent variables affecting behavioral response to the human intruder test in infant rhesus monkeys (Macaca mulatta). *American Journal of Primatology* 75:314-323.
5. Kalin NH & Shelton SE (1989) Defensive behaviors in infant rhesus monkeys: environmental cues and neurochemical regulation. *Science (New York, N.Y.)* 243(4899):1718-1721.
6. Golub MS, Hogrefe CE, Widaman KF, & Capitanio JP (2009) Iron deficiency anemia and affective response in rhesus monkey infants. *Developmental psychobiology* 51(1):47-59.
7. Kinnally EL*, et al.* (2010) Serotonin pathway gene-gene and gene-environment interactions influence behavioral stress response in infant rhesus macaques. *Development and psychopathology* 22(1):35-44.
8. Dobin A*, et al.* (2013) STAR: ultrafast universal RNA-seq aligner. *Bioinformatics (Oxford, England)* 29(1):15-21.
9. Evans C, Hardin J, & Stoebel DM (2018) Selecting between-sample RNA-Seq normalization methods from the perspective of their assumptions. *Briefings in bioinformatics* 19(5):776-792.

10. Cole SW, Shanahan MJ, Gaydosh L, & Harris KM (2020) Population-based RNA profiling in Add Health finds social disparities in inflammatory and antiviral gene regulation to emerge by young adulthood. *Proceedings of the National Academy of Sciences of the United States of America* 117(9):4601-4608.

11. Cole SW, Yan W, Galic Z, Arevalo J, & Zack JA (2005) Expression-based monitoring of transcription factor activity: The TELiS database. *Bioinformatics (Oxford, England)* 21(6):803-810.

12. Eisenberg E & Levanon EY (2013) Human housekeeping genes, revisited. *Trends in genetics : TIG* 29(10):569-574.

13. Shi L*, et al.* (2008) The balance of reproducibility, sensitivity, and specificity of lists of differentially expressed genes in microarray studies. *BMC Bioinformatics.* 9(Suppl 9):S10.

14. Cole SW, Galic Z, & Zack JA (2003) Controlling false-negative errors in microarray differential expression analysis: a PRIM approach. *Bioinformatics (Oxford, England)* 19(14):1808-1816.

15. Witten DM & Tibshirani R (2007) A comparison of fold-change and the t-statistic for microarray data analysis. (Stanford University Technical Report, Stanford), pp 1-17.

16. Fredrickson BL*, et al.* (2015) Psychological well-being and the human conserved transcriptional response to adversity. *PloS one* 10(3):e0121839.

17. Efron B & Tibshirani RJ (1993) *An introduction to the bootstrap* (Chapman & Hall, New York).

18. Flygare S*, et al.* (2016) Taxonomer: an interactive metagenomics analysis portal for universal pathogen detection and host mRNA expression profiling. *Genome biology* 17(1):111.

19. Chun K*, et al.* (2017) Social regulation of the lymph node transcriptome in rhesus macaques (Macaca mulatta). *Psychoneuroendocrinology* 76:107-113.
